# Integrating *Vibrio natriegens* for Photon Manipulation in Living Lighting Devices

**DOI:** 10.1002/adma.202514435

**Published:** 2025-12-26

**Authors:** Stephanie Willeit, Maurice Hädrich, Philippe‐Quentin Liss, Valeria Rodriguez Espinoza, Nicklas Maximilian Foerster, Bastian Blombach, Rubén D. Costa

**Affiliations:** ^1^ Technical University of Munich Campus Straubing for Biotechnology and Sustainability, Chair of Biogenic Functional Materials Straubing Germany; ^2^ Technical University of Munich Campus Straubing for Biotechnology and Sustainability, Microbial Biotechnology Straubing Germany; ^3^ Technical University of Munich SynBiofoundry@TUM, Campus Straubing for Biotechnology and Sustainability Straubing Germany

**Keywords:** bacteria‐hybrid light‐emitting diodes, engineering living materials, fluorescent proteins, photon management, *Vibrio natriegens*

## Abstract

Photon manipulation with bacteria is an emerging field in photonics (lasers, bio‐imaging, and cell‐sensing) and optoelectronics (bacterial‐hybrid light‐emitting diodes (BaHLEDs) and photovoltaics). In BaHLEDs, living photon down‐conversion filters based on the direct use of bacteria in optically desirable hydrophobic and/or waterless coatings have not been realized yet. Herein, we put forward a simple concept: the engineering of fluorescent *Vibrio natriegens* (*V. natriegens*) that enable easy‐to‐prepare, untreated bacteria‐silicone filters for the first red‐emitting BaHLEDs. More specifically, we have rationalized genetic and material engineering tools to optimize i) the protein production time with a 1.7‐fold enhanced volumetric productivity compared to *E. coli* with the same spectroscopic quality for the fluorescent protein (FP) DsRed, ii) the straightforward fabrication of highly emissive and stable *V. natriegens*‐silicones, and iii) device reproducibility and performance to reach competitive devices with stabilities ranging from a few days up to weeks depending on device working conditions and architectures. Overall, this work sets in bacterial photon down‐conversion using *V. natriegens* directly as a viable and more straightforward concept toward further advances in living lighting applications.

## Graphical abstract

Rationalization of genetic and material engineering tools has enabled the engineering of fluorescent Vibrio natriegens (V. natriegens) to fabricate straightforward, highly emissive and stable V. natriegens‐silicones color filters for the first red‐emitting bacteria‐hybrid light‐emitting diodes. This pinpoints V. natriegens as a high‐performance protein production platform to advance bacteria optoelectronics, in general, and bacteria photon down‐conversion filters for lighting, in particular.

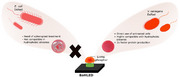

## 1 Introduction

The concept of engineered living materials (ELMs) is an emerging field for the development of responsive, adaptive, self‐healing, eco‐friendly, and genetically programmed functional materials [1, 2]. Synthetic biology, microbiology, and materials science are already working at full speed to successfully advance biomedical applications (wound healing, drug delivery, etc.) [1, 2, 3], in situ bio‐sensing [3], living electronics (sensors, neurological interfaces, etc.) [4], bio‐hybrid soft robotics [5, 6], and, more recently, living optoelectronics and photonics (photovoltaics, lasers, photonic wires, etc.) [4, 7, 8], among others. Thus, ELMs promise to help toward a sustainable biologization of our current technologies, opening new horizons at the interface between our technological needs and living matter [1, 2, 3, 4, 5, 6, 7, 8].

In the field of living photon amplification, a pioneering work was reported by Gather and Yun et al. who demonstrated lasers based on a single cell that produces fluorescent proteins (FPs) as gain medium [9], while the resonator structure can be located either outside the cell (Fabry–Pérot cavity) or inside as a result of the refractive index difference between the cell and the surrounding medium [10]. First uses have already been disclosed in cell tagging/tracking [11, 12] and intracellular sensing [12]. However, first attempts to use bacteria (*E. coli*) as photon down‐converting materials have resulted in a low viability (bacterial lysis and/or aggregation) and respective high scattering and auto‐fluorescence quenching phenomena upon preparation of polymer color filters [1, 2, 4, 7, 8, 13]. This was recently circumvented by forming green‐emitting spheroplasts with i) >90% scattering reduction, as the membrane wall and the cell size were significantly reduced by the surrounding maltodextrin (high osmotic pressure), ii) a high density of green‐emitting FPs at the cytoplasm, and iii) an easy recyclability from the hydrophilic poly(vinyl alcohol) coatings [13]. This enabled the preparation of bacterial‐hybrid light‐emitting diodes (BaHLEDs) integrating treated *E. coli* (spheroplasts)‐PVA photon down‐conversion filters in lighting devices, realizing a green emission with similar stabilities to the respective purified FPs embedded in the same polymer‐based filters. Thus, the concept of living photon filters could be more advantageous than FP‐polymers, as costly FP purification is not required, and the bacteria‐polymer filters could potentially be adaptive to external changes [13]. Thus, BaHLEDs could also represent a biogenic alternative to replace the non‐sustainable rare‐earth (Ce:YAG) or toxic (Cd‐based quantum dots) photon down‐converting filters used in current white LEDs, as mandated by the EU/US Lighting and Material 2030 roadmap [14, 15, 16, 17].

Although the BaHLED concept offers great hopes, the formation and stabilization of spheroplasts (treated *E. coli*) in hydrophilic polymers to fabricate suitable living photon down‐converting filters for illumination purposes is costly and time‐consuming [13]. Finally, the archetypal *E. coli* also exerts limitations related to i) its moderate biomass production and growth, and ii) upscale bio‐transformation (toxicity) [18, 19, 20, 21]. To address these challenges, this work puts forward the idea of engineering fluorescent *V. natriegens* as a more versatile bacterial platform to further advance the concept of BaHLEDs. The motivation is three‐fold: i) its outstanding bio‐production with the swiftest growth rate among all established bacteria for heterologous expression with a doubling time of <10 min [22, 23], a versatile metabolic capacity enabling to use many substrates as carbon and energy sources [20, 24, 25], and a tendency for high level protein production [20, 26, 27], ii) the still growing toolbox for genetic engineering its metabolism [25, 28, 29] with only few works showing the production of, for example, enhanced green (EGFP) and yellow FP (EYFP) markers/reporters [22, 27, 28, 30, 31, 32], while the protein quality (folding to maximize photoluminescence quantum yields (ϕ)) as well as versatility aspects for any FP expression have not been deeply investigated yet [27, 28], and iii) its capsular and extracellular polysaccharide (EPS) for biofilm formation and its distinct surface membrane morphology adapted to high osmotic pressures as a halophilic marine bacterium allows physical/chemical microenvironment control around the cells, affecting chemical biotransformation rates and resilience, as examples [33, 34, 35, 36].

Herein, we have rationalized genetic tools and optimized growth protocols to produce the first red‐emitting *V. natriegens* with excellent expression of the red‐emitting *Discosoma* Red Fluorescent Protein (DsRed). What is more, *V. natriegens* proved to be surprisingly compatible with a hydrophobic silicone matrix, in which *E. coli* tends to form large visible aggregates that are not suitable for device application. This could be related to its distinct cell surface morphology and EPS composition. The *V. natriegens*‐silicone filters are, indeed, visually homogeneous, regardless of the shape (films and hemispheres), similarly bright as the respective FPs in the cells, and recultivable in media. Finally, these living filters were applied to prepare the first red‐emitting BaHLEDs, showing stabilities ranging from a few days up to weeks depending on the device working conditions and architectures. Overall, this work pinpoints *V. natriegens* not only as a high‐performance platform for recombinant protein production, but also as a highly promising platform to advance living optoelectronics, in general, and living photon down‐conversion filters for lighting, in particular.

## 2 Results and Discussion

### 2.1 Red‐Emitting *V. natriegens*

The optimized expression of DsRed in *E. coli* is carried out by first cultivating the cells in round flasks at 200 rpm at 30°C to promote cell growth for *ca*. 2 h, followed by isopropyl β‐D‐1‐thiogalactopyranoside (IPTG) induction and a temperature reduction down to 16°C for optimum FP expression over 48 h. Finally, an increase of temperature up to 30°C for 3 h was applied to ensure a successful chromophorylation of the FPs (see experimental section for details). As a quick check of the FP quality for photon down conversion purposes, the photoluminescence quantum yield (ϕ) of the bacteria pellet is typically determined. This leads to intense red *E. coli* cell pellets with a ϕ of *ca*. 60% that further enhances to the expected *ca*. 80% after extraction and purification of the DsRed protein [37]. Thus, the difference in ϕ values might be related to scattering and refractive index mismatch between the samples.

**FIGURE 1:**
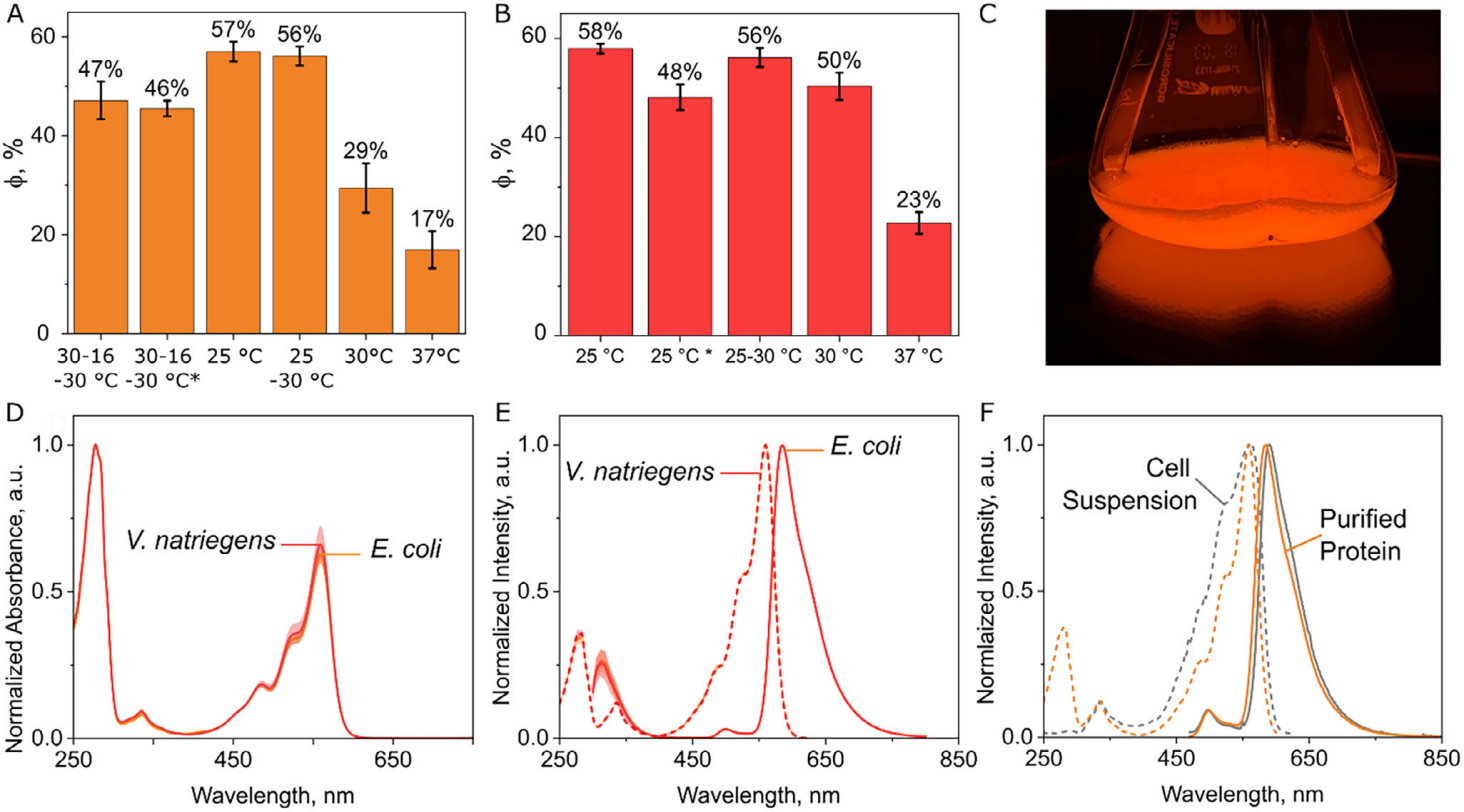
Screening of cultivation conditions in independent triplicates with respect to the changes of the ϕ of DsRed pelleted cells using cultivation times of (A) 48 h and (B) 24 h at different temperatures. The comparison was done in baffled and non‐baffled flasks (*). (C) Picture of a *V. natriegens* cell culture after optimizing cultivation temperature and incubation time. (D) Absorbance (E) emission (solid line; λ_ex_ = 280 nm) and excitation (dashed line; λ_em_ = 630 nm) spectra of purified DsRed produced by *E. coli* and *V. natriegens* (n = 3 batches). (F) Emission (solid line; λ_ex_ = 450 nm) and excitation (dashed line; λ_em_ = 640 nm) spectra of purified DsRed (orange) and *V. natriegens* cells expressing DsRed in suspension (grey).

Next, we used the optimized LBv2 medium protocol for *V. natriegens Vmax^TM^X2* [33, 38] and a plasmid without codon optimization for protein expression in this organism. In detail, the DsRed gene was carried by a pET29b(+) vector containing a kanamycin resistance (Figure S1; see experimental section for details). As first attempts for cultivation, we adapted the above optimized *E. coli* protocol to a timeframe of up to 48 h and IPTG induction at OD_600_ of 0.4–0.6 (see experimental section for details), reaching a promising red *V. natriegens* culture (Figure 1A–C). Unfortunately, the ϕ value of the pellet was around 45% (Figure 1A), indicating a poor protein quality production (vide supra). This is not surprising, since a temperature range between 25°C and 37°C is commonly best suitable for the recombinant protein production of *V. natriegens* Vmax^TM^X2 [27, 38, 39]. In addition, this should not be related to the oxidation reactions during chromophore maturation [40, 41, 42], as similar results were obtained using both, baffled and non‐baffled flasks (* in Figure 1A,B), resulting in different O_2_ concentration and diffusivity upon cultivation [43, 44]. Thus, the low protein quality was tentatively attributed to the inefficient metabolic activity at 16°C of *V. natriegens* [27, 28, 39]. Thus, we turned to investigate the DsRed quality production at 25°C, 30°C, and 37°C for both, 24 and 48 h of incubation. At short cultivation times (Figure 1A,B), the quality of the DsRed reached an optimum value of *ca*. 60% at 25°C, while it progressively reduced to around 50% and 23% at 30°C and 37°C, respectively. This suggests that protein misfolding is promoted with the increased temperature, as already noted for recombinant protein expression with *E. coli* [45, 46]. In addition, as expected by the oxygen dependency of the chromophore maturation [40, 41, 42], the ϕ values of the pellets reduced to 48% using non‐baffled flasks at 25°C for 24 h (* in Figure 1B) [27]. Finally, long cultivation times nicely confirmed the above trend with even more dramatic decrease of the ϕ from 30°C on (Figure 1A,B). This could be related to protein aggregation that easily occurs at high protein overexpression, protein misfolding, and/or protease‐driven degradation upon stress [47, 48].

In order to determine the quantity and quality of DsRed production in both microorganisms in their respective optimized production protocols, the FP were extracted and purified using a common protocol for both bacteria (see Supporting Information for details). Hereby, the quantity and quality of the DsRed can be compared for both microorganisms (Figure 1D,F and Tables 1 and 2). On one hand, both bacterial deliver a similar product concentration of 119 ± 21 mg L^−1^ (*E. coli*) and 102 ± 19 mg L^−1^ (*V. natriegens*), while the volumetric productivity of *V. natriegens* is 1.7‐fold enhanced compared to that of *E. coli* (Table 2), supporting that *V. natriegens* is indeed a high‐performance platform for FP production. On the other hand, the absorption, emission spectra (λ_ex_ = 280 and 450 nm), and excitation spectra (λ_em_ = 630 nm) are similar to those of the maturated red‐emitting form of the chromophore of DsRed (Figure 1D,E). In addition, a low‐intense emission band located at 500 nm associated to the not fully maturated green‐emitting form of the chromophore is also noted (Figure 1E) [37, 42, 49]. Importantly, the ratio between the intensity of the green and red emission bands is the same for *V. natriegens* cells with DsRed and the purified DsRed produced by both *V. natriegens* and *E. coli*, indicating that the chromophore maturation is similar for the above protocol. This is also confirmed by the alike excited‐state lifetime (τ) and ϕ values (Table 1). Finally, the fine vibrational structure of the excitation spectra also suggests the lack of protein agglomeration in the cytoplasm (Figure 1F), while the small differences could be attributed to different refractive index mismatch, pH, viscosity, and O_2_ content changes present in the cytoplasm environment of the bacteria. Hence, we can conclude that the quality of the DsRed expression in *V. natriegens* is highly reproducible and comparable to that of the optimized *E. coli*, with the advantage of a 2‐fold reduced protein production time and 1.7‐fold enhanced volumetric productivity. Thus, the engineered red‐emitting *V. natriegens* could be suitable for photon down‐conversion purposes if this survives the fabrication of polymer coatings.

**TABLE 1:** Photoluminescence parameters of purified DsRed produced by *E. coli* or *V. natriegens* in solution (PBS buffer, pH 7.4), cell suspension, cell pellet, and silicone‐embedded *V. natriegens* cells. These values are the average (mean ± S.D.) of at least three individual production batches for each *E. coli* and *V. natriegens*.

	Pur. protein *E. coli*	Pur. protein *V. natriegens*	Cell suspension	Cell pellet	Silicone
λ_ex_[nm]^a^	559	559	560	560	562
Λ_em_[nm]^b^	584	584	590	600	597
ф[%]^c^	80.1 ± 0.2	79.1 ± 0.4	66.8 ± 2.0	58.3 ± 1.1	58.1 ± 0.5
τ_450nm_[ns]^d^	3.84 ± 0.03	3.82 ± 0.02	3.65 ± 0.02	4.22 ± 0.04	4.12 ± 0.23

^a^ Maximum excitation wavelength;

^b^ Maximum emission wavelength;

^c^ Photoluminescent quantum yield at λ_ex_ = 520 nm;

^d^ Excited‐state lifetime atmaximum emission wavelength λ_ex_ = 450 nm.

**TABLE 2:** Comparison of product concentration and volumetric productivity of DsRed between *E. coli* (pET29b(+) DsRed) and *V. natriegens* (pET29b(+) DsRed). These values are the average (mean ± S.D.) of three individual production batches for each *E. coli* and *V. natriegens*.

Microorganism	Product Concentration [mg L^−1^]	Volumetric Productivity [mg L^−1^ h^−1^]
*E. coli* BL21 (pRT29b(+) DsRed)	118.90 ± 21.18	2.48 ± 0.44
*V. natriegens* Vmax^TM^ X2 (pRT29b(+) DsRed)	101.89 ± 19.31	4.25 ± 0.80

### 2.2 Red‐Emitting *V. natriegens*‐Silicone Filters

As aforementioned, *V. natriegens* was not only chosen by the high growth rate and excellent FP production time and quality (vide supra), but also by the opportunity to capitalize on its unique cell surface morphology, displayed by an altered fatty acid composition of the membrane compared to *E. coli*, and the ability to produce EPS that could increase the performance in polymer matrices after noting the key role of maltodextrin in spheroplast‐based devices [13, 33, 35, 36]. Thus, our hypothesis was that *V. natriegens* could feature an enhanced viability/compatibility with arbitrary encapsulating materials, in general, and hydrophobic silicone matrices more suitable for lighting devices [50, 51], in particular. In this context, we turned our attention to thin self‐standing films using a transparent bio‐compatible silicone matrix (ELASTOSIL) that features a vacuum‐/irradiation‐free and quick ambient curing process and keeps the optical features with a reasonable water‐content (<20% v/v). In short, 50 mg of both *E. coli* and *V. natriegens* cell pellets were mixed with the silicone matrix component upon gentle manual stirring to reach a homogeneous distribution, while the silicone hardener component was subsequently added. The mixture was placed in the desired mold (films of 2.5 cm × 2.5 cm × 2 mm and hemispheres of 4 mm height and 10 mm diameter) to cure over time (see experimental section for details).

**FIGURE 2:**
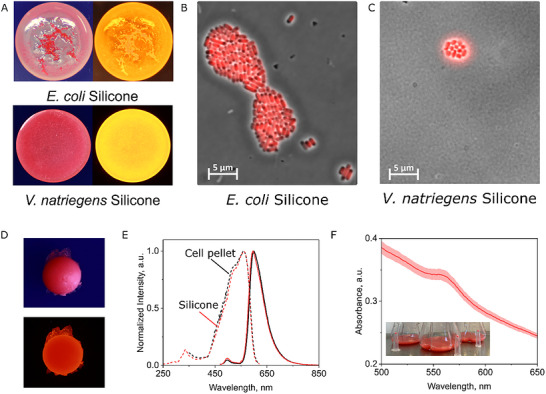
(A) *E. coli* (top) and *V. natriegens* (bottom) based silicone thin films (2.5 cm × 2.5 cm × 2 mm) under ambient (left) and excited with a blue‐transilluminator (right); please note that the yellow color is related to the combination of emission from the sample and the excitation system as the coatings are thin. (B) Microscopic picture of *E. coli* and (C) *V. natriegens* cells highly diluted in silicone thin‐films. (D) *V. natriegens*‐silicone hemisphere shaped filters under ambient (top) and excited with a blue‐transilluminator (bottom); please note that the red color is now clearly visible due to the sample dimensions (4 mm height and 10 mm diameter). (E) Emission (solid line; λ_ex_ = 450 nm) and excitation (dashed line; λ_em_ = 640 nm) spectra of pellet (black) and hemisphere‐shape silicone filter of *V. natriegens* cells expressing DsRed (red). (F) Absorption spectrum of the recultivated, silicone‐embedded *V. natriegens* cells after 3 days of cultivation at 25°C (inset picture).

As anticipated, the *E. coli* thin‐films resulted in agglomerated and non‐homogeneous distribution (Figure 2A), while the mixture of silicone and *V. natriegens* exhibited a homogeneous appearance to the naked eye (Figure 2A). This was confirmed by both optical microscopy and emission spectroscopy techniques. On one hand, microscope analysis suggests that the bacteria‐silicone coatings are better described as a heterogeneous dispersion of large aggregated bacteria in *E coli*, while it is a homogeneous dispersion of small cell aggregates in *V. natriegens* (Figure 2B,C). On the other hand, the emission/excitation spectra and figures‐of‐merit did not significantly change upon comparing *V. natriegens* in pellets and silicone filters (Figure 2E and Table 1). In detail, they featured ϕ and τ values of 58% and 4.12 ns associated with very similar emission and excitation spectra that are reproducible over independent *V. natriegens* batches (Supporting Information for details, and Figure S2). While the spectroscopic features are similar to those in cell pellets (Figure 2E and Table 1), the differences with those in solution could be related to changes in the pH, viscosity, refractive index, and O_2_ content in the cytoplasm of *V. natriegens* under coating and pellet preparation (vide supra). Finally, the resilience and living features of *V. natriegens*‐silicone were confirmed by testing the survival behavior of *V. natriegens* and re‐expression capability of DsRed upon recultivating the *V. natriegens*‐silicone coatings in media (Figure 2F). The readily fresh coatings were placed into liquid media, and the FP expression was induced following the regular protocol (see experimental section for details). They evolved in deep red liquid cultures with a high inner‐protein content that is clearly visible in the absorbance spectra of the cultures (Figure 2F). Overall, these findings show the potential of forming a living photon down‐converter by encapsulating *V. natriegens* in a hydrophobic silicone matrix with excellent photoluminescent features for device application.

### 2.3 Red‐Emitting *V. natriegens*‐Hybrid Light‐Emitting Devices

**FIGURE 3:**
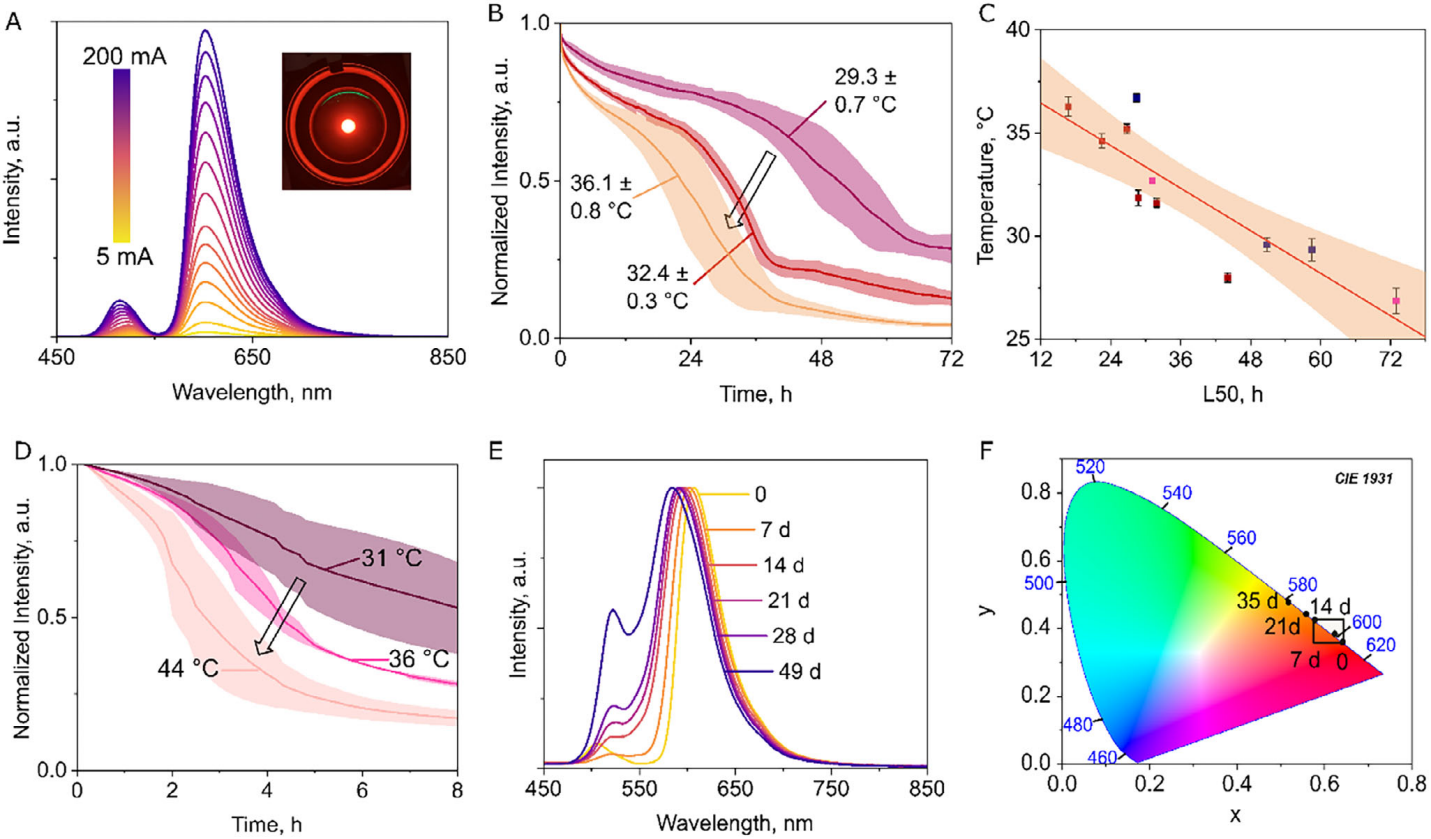
(A) Emission spectra of *V. natriegens*‐devices upon increasing applied current from 5 mA to 200 mA, including a picture of an operating BaHLED at 200 mA with a 520 nm LED chip. (B) Averaged normalized intensities of devices operating at similar temperatures (the shadow area refers to the associated averaged curve of the replicates shown in the Supporting Information Figures S5–S8). (C) The correlation of maximum device temperature and stability (L50) of three independent batches, as indicated by the different colors (red, pink, blue), and several representatives are shown in Figures S5–S8. (D) Normalized intensity of fluorescence loss upon incubation of *V. natriegens*‐silicones at various temperatures (the shadow area refers to the associated averaged curve of five replicates each). (E) Emission spectra of *V. natriegens*‐devices in a remote configuration over time. (F) *x/y* CIE color coordinate changes of *V. natriegens*‐devices in a remote configuration over time, highlighting the color stability of *ca*. 14 days (black square).

BaHLEDs were fabricated by placing the above hemisphere‐shaped *V. natriegens*‐silicone filters in direct contact with the unmodified commercial LED (520 nm, WINGER, 1 W). Here, we analyzed three independent batches of *V. natriegens*‐silicone coatings using different conditions/architectures to determine the impact of the coating preparation (i.e., intracellular DsRed concentration, pellet formation, and water content) and operation conditions (i.e., excitation photon powers from 80 to 120 mW/cm^2^ and different coating temperatures; Figure S3) on the device performance. At first, all the devices showed an almost complete photon down‐conversion efficiency (>95%; Figure S4) with a clear dominant red emission band centered at 590 nm (*x*/*y* CIE color coordinates of *ca*. 0.61/0.37) that perfectly resembles the photoluminescence of the filters regardless of the applied current (Figure 3A). In addition, a gradual increase of the luminance with the applied current, reaching maximum values of *ca*. 35 000 cd/m^2^ at an applied current of 200 mA was noted (Figure S4).

To determine the device stability, the devices were operated at incident photon excitation power spanning from 80 to 120 mW/cm^2^, resulting in an increase of the *V. natriegens*‐silicone coatings temperature going from *ca*. 28°C to 37°C (see Supporting Information for details Figure S3). For each excitation/temperature condition, the emission intensity of the conversion band and the overall device chromaticity were monitored (Figure 3B; see Supporting Information for details Figures S5–S8). Here, the emission intensity decay is divided into three parts: i) a first decay that goes hand‐in‐hand with the temperature rise of the filter (i.e., temperature‐induced emission quenching) with respect to intensity loss and timing (see Figures S5–S8), ii) a slow linear intensity decay related to the photo‐bleaching of the DsRed at the respective maximum working temperature, and iii) a sharp decay reaching a 50% intensity loss compared to the initial intensity (L50 or device stability). Over the span of these measurements, the LED intensity also increases, leading to a color corruption from red to yellowish orange devices close to the starting point of the third regime (solid line in Figures S5–S8). Much more importantly, there is a linear relationship between the L50 values and the working temperatures (Figure 3C). Here, the device stability spans from *ca*. 18 h (Figure S7) up to *ca*. 72 h (Figure S8) at operating temperatures of 37°C and 28°C, respectively. This trend is derived from the analysis of three independent batches with several replicates that should average differences coming from the intracellular DsRed concentration, pellet formation, and/or water content.

While irradiation stress should not activate oxidative stress upon exciting DsRed [52], *V. natriegens* is very sensitive to temperature changes as previously studied [27, 28, 53, 54] as well as there are no detailed studies about visible irradiation‐induced stress and/or metabolic changes on *V. natriegens* up to date, especially the applied 520 nm excitation source [55, 56, 57, 58]. Thus, we decided to decouple both temperature and irradiation factors. On one hand, the emission intensity loss of a 100‐fold diluted *V. natriegens*‐silicone coatings incubated at 31°C, 36°C, and 44°C was monitored (Figure 3D). At constant 44°C and 36°C, the slope of the emission intensity decay features a shallow gradient during the first 1.5 h, followed by an exponential decay, reaching a 50% intensity loss at 2 and 4 h, respectively. In contrast, samples at 31°C featured a slow linear emission decay, reaching the 50% intensity loss after 8 h. Thus, the temperature window for the device operation seems to be narrow and might exert a significant impact on the device stability. On the other hand, we carried out the stability experiments with the original *V. natriegens*‐silicone coatings using the remote device configuration (i.e., 2 cm distance from the LED) that leads to a low incident photon excitation of 2 mW/cm^2^ operating at *<*25°C, reaching color stability over 14 days (Figure 3E,F). This indicates that *V. natriegens*‐silicones might be less sensitive to the applied irradiation than to the working temperature.

## 3 Conclusions

This work discloses a simple concept to advance the emerging field of living photon manipulation for illumination purposes (BaHLEDs) by capitalizing on the well‐known fast growing, efficient metabolism for FP expression, the better adaptability of *V. natriegens* against osmotic stress environments, and its ability to produce capsular and extracellular polysaccharides. Compared to treated *E. coli* (spheroplasts) BaHLEDs [13], *V. natriegens* surprisingly enables the straightforward (no need for additives, ambient processing conditions, and hydrophobic encapsulating materials) use of the untreated bacteria in a silicone matrix, keeping ϕ and recultivation features. What is more, protocols for the efficient and faster FP expression in *V. natriegens* compared to *E. coli* (1.7‐fold enhanced volumetric productivity) have been optimized for the first red‐emitting *V. natriegens* with DsRed without compromising its emission figures‐ of‐merit. Finally, the easy handling of *V. natriegens*‐silicone coatings has resulted in performing devices with respect to stabilities between days‐weeks, depending on the device working conditions and architecture. This device stability is in line with the literature of FP‐/spheroplast‐based lighting devices [13, 59, 60, 61]. However, the major limitation is the narrow temperature/excitation power windows when operating *V. natriegens*‐BaHLEDs.

Overall, this work heralds that *V. natriegens* shines not only as a highly promising microbe for large‐scale bioproduction, but also as a robust and versatile bacterial platform to engineer living photon manipulation materials for photonics and lighting applications. At present, we are focused on two aspects. On the one hand, the engineering of new fluorescent and thermal resistance *V. natriegens* strains that are viable in other rigid polymer matrices, hydrogel, and multilayered coatings, aiming to set in tools for the integration of diverse FPs and external triggers for protein production in the genome. We hope to enhance the resilience toward temperature and irradiation stress and/or to demonstrate adaptability. On the other hand, the demonstration of the scalability in mid‐and large‐size bioreactors to provide an accurate life‐cycle and cost‐effective analysis to assess environmental footprint and sustainability.

## Conflicts of Interest

The authors declare no conflicts of interest.

## Supporting information




**Supporting File**: adma71821‐sup‐0001‐SuppMat.docx

## Data Availability

The data that support the findings of this study are available from the corresponding author upon reasonable request.
