# Supplementary material for: Integrating Vibrio natriegens for Photon Manipulation in Living Lighting Devices
Source: Adv Mater. 2025 Dec 26;38(34):e14435. doi: 10.1002/adma.202514435 (PMC13274690; doi:10.1002/adma.202514435)
Supplement: Supplementary file 1 — Supporting File: adma71821‐sup‐0001‐SuppMat.docx [file ADMA-38-e14435-s001.docx]

Supplementary Information

**Integrating *Vibrio natriegens* for photon manipulation in living lighting devices**

*Stephanie Willeit,^a^ Maurice Hädrich,^b,c^ Philippe-Quentin Liss,^a^ Valeria Rodriguez Espinoza,^b^ Nicklas Maximilian Foerster,^b^ Bastian Blombach^b,c^* and Rubén D. Costa^a^**

^a^Technical University of Munich, Campus Straubing for Biotechnology and Sustainability, Chair of Biogenic Functional Materials, Schulgasse, 22, Straubing 94315, Germany.
E-mail: [ruben.costa@tum.de](mailto:ruben.costa@tum.de)

^b^Technical University of Munich, Campus Straubing for Biotechnology and Sustainability, Microbial Biotechnology, Uferstraße, 53, Straubing 94315, Germany. E-mail: bastian.blombach@tum.de

^c^Technical University of Munich, SynBiofoundry@TUM, Campus Straubing for Biotechnology and Sustainability, Straubing, Germany.

**Methods**

*FP production and purification in V. natriegens and E. coli*

The pET29b(+) DsRed plasmid, encoding DsRed under the control of the T7-LacO promoter, was purchased from Twist Bioscience HQ, USA and further amplified and isolated from an *E. coli* BL21 DE3 overnight culture (Luria Bertani (LB) medium, pH 7.4, 30 °C, 200 rpm). The insert was verified by sequencing (Eurofins, Constance, Germany) before being introduced into competent *V. natriegens* Vmax X2^[1]^ by electroporation as described previously.^[2,3]^ DsRed was subsequently expressed in *V. natrigens* Vmax^TM^ X2^[1]^ using the described pET29b(+) plasmid. *V. natriegens* Vmax™ X2 facilitates efficient protein production via a chromosomally integrated IPTG-inducible T7 RNA polymerase system. For the cultivation of *V. natriegens* Vmax^TM^ X2^[1]^, LBv2 medium^[4]^ supplemented with 200 μg mL^−1^ of kanamycin was used. The 5 mL preculture was inoculated overnight at 37 °C and 180 rpm. The FP expression experiments were performed in 500 mL baffled or non-baffled shaking flasks containing 50 mL LBv2 medium at various temperatures at 200 rpm. The inoculation was done performed at OD_600_ 0.1, followed by an induction at OD_600_ 0.4-0.6 with 1 mM isopropyl β-D-1-thiogalactopyranoside (IPTG). The incubation time was either 24 or 48 h after induction. The optimized protocol for FP expression in a 500 mL baffled shaking flasks containing 50 mL LBv2 medium at 25 °C at 200 rpm. The inoculation was performed at OD_600_ 0.1, followed by an induction at OD_600_ 0.4-0.6 with 1 mM IPTG. The incubation time was 24 h after induction.

The pET29b(+) DsRed plasmid was transformed into competent *E. coli* BL21 DE3 by electroporation. *E. coli* was cultivated in Luria Bertani (LB) media pH 7.4 containing 50 μg kanamycin mL^−1^. The pH was adjusted with 5 M NaOH. The preculture was inoculated and cultivated at 30 °C and 200 rpm overnight. On the following day, the main culture was cultivated in 2 L shaking flasks containing 300 mL of LB media. For the optimized expression of DsRed in *E. coli* the inoculation was done at an OD_600_ of 0.1, first cultivating the cells at 30 °C 200 rpm to promote cell growth for *ca*. 2 h, until an OD_600_ of 0.4-0.6 followed by 1 mM IPTG induction and a temperature reduction down to 16 °C for 48 h. Finally, an increase of temperature up to 30 °C for 3 h was performed to ensure a successful chromophorylation of the FPs.

For recultivation, fresh silicone hemisphere-shaped filters containing *V. natriegens* Vmax X2^[1]^ with expressed DsRed were disinfected and cut in small pieces before the cultivation in LBv2 media ^[4]^ containing 200 μg kanamycin mL^−1^. The 10 mL preculture was inoculated with the small material pieces overnight at 30 °C and 200 rpm. The expression experiments were performed in 500 mL baffled shaking flasks containing 50 ml LBv2 media at 25°C and 200 rpm over the weekend (3 days). The inoculation was done at OD_600_ 0.1, followed by an induction at OD_600_ 0.4-0.6 with 1 mM IPTG.

In order to purify the FPs, the cell pellets were frozen at -20 °C for at least 12 hours. The thawed cell pellet was then resuspended in an extraction buffer (50 mM Tris, 300 mM NaCl, 1 % Triton™ X-100, 5 % glycerol, 5 mM EDTA, 0.3 mg lysozyme mL^-1^) and incubated for 1 hour at room temperature and 20 rpm overhead rotation. The cells were then lysed by tip sonication (8 min of sonication in 1 s long pulses with 3 s pause in between them; Tip-Sonicator CL-334, Fisher Scientific). The FPs were subsequently purified via affinity chromatography using the Äkta HighPure LC system with a HisTrap^TM^ HP column followed by storage of the purified protein solution in the dark over night at room temperature before desalting using a HiPrep^TM^ desalting column. The purified protein solution was then quantified using a UV–vis-2600i spectrophotometer (Shimadzu) from 250 to 900 nm with medium scan speed, a data interval of 1 nm, and 1 nm slit width.

The DNA sequence of DsRed is:

ATGCACCATCATCATCATCATGGTTCTGGCCGCTCATCGAAAAACGTCATTAAAGAATTTATGCGCTTTAAAGTACGCATGGAAGGCACTGTTAACGGACACGAATTTGAAATTGAAGGCGAGGGCGAGGGTCGGCCGTACGAAGGTCACAATACAGTCAAGCTTAAGGTTACGAAAGGAGGGCCGCTCCCATTCGCATGGGACATACTGTCACCGCAGTTTCAGTATGGGAGCAAAGTGTACGTAAAACATCCAGCTGATATCCCGGATTATAAGAAATTATCATTTCCGGAGGGTTTTAAGTGGGAACGTGTGATGAATTTCGAAGATGGAGGAGTTGTCACAGTCACCCAGGATTCCTCACTGCAGGACGGATGTTTTATTTATAAAGTGAAATTCATAGGTGTCAATTTTCCTTCTGATGGACCGGTTATGCAGAAAAAAACCATGGGATGGGAAGCATCGACTGAACGCTTGTATCCGCGTGATGGCGTTCTGAAAGGTGAAATTCATAAAGCGTTGAAATTAAAGGATGGCGGTCACTATCTGGTTGAATTTAAATCTATATATATGGCGAAAAAGCCCGTACAGCTTCCGGGGTATTATTATGTAGATAGTAAATTGGACATTACCTCACATAATGAAGATTACACAATCGTGGAGCAATATGAAAGAACGGAAGGACGCCACCATCTGTTCCTGTAA

The reported DNA sequence is translated to the following amino acid sequence:

MHHHHHHGSGRSSKNVIKEFMRFKVRMEGTVNGHEFEIEGEGEGRPYEGHNTVKLKVTKGGPLPFAWDILSPQFQYGSKVYVKHPADIPDYKKLSFPEGFKWERVMNFEDGGVVTVTQDSSLQDGCFIYKVKFIGVNFPSDGPVMQKKTMGWEASTERLYPRDGVLKGEIHKALKLKDGGHYLVEFKSIYMAKKPVQLPGYYYVDSKLDITSHNEDYTIVEQYERTEGRHHLFL*

*Characterization techniques*

Absorption spectra of FP solutions or cell suspensions were recorded with a UV–vis-2600i spectrophotometer (Shimadzu) from 250 to 900 nm, or 400 – 800 nm, respectively, with medium scan speed, a data interval of 1 nm, and 1 nm slit width. Photophysical studies were carried out at ambient conditions using an FS5 spectrofluorometer (Edinburgh Instruments) with the SC-05 module for liquid samples and the SC-10 module for solid samples. A time-correlated single photon-counting module was used to determine τ and adjusted to an exponential decay fit with Origin 2021b (OriginLab Corporation, Northampton, MA, USA). To calculate the average lifetime for each sample, the following equation was used:

$<\tau> = \frac{\sum a_{i}\tau_{i}^{2}}{\sum a_{i}\tau_{i}}$

where a_i_ (λ) are the amplitude fractions and τ_i_ are the lifetimes.^[5]^ The photoluminescence quantum yield (φ) was measured using a Quantaurus-QY Absolute PL quantum yield spectrometer (Hamamatsu Photonics). Thermocycler-based Modulated Scanning Fluorimetry at different incubation temperatures was performed and analyzed with the Thermocycler CFX96 Touch Real-time PCR System (Bio-Rad). The program is based on constant heating (temperatures from 30 °C to 45 °C) to measure the progressive loss of fluorescence of highly diluted DsRed *V. natriegens* silicone filters. Fluorescent microscopy images were taken using a Zeiss Axio Observer Z1 (Germany) with phase contrast and 100× objective, and Axiocam 503 and ZenBlue 3.2

*Device preparation and characterization*

ELASTOSIL^®^ RT 604 A and B were purchased from WACKER. 50 mg of both, *E. coli* and *V. natriegens* cell pellets, were mixed with 150 µL of the silicone matrix component A upon manual stirring to reach a homogenous distribution. Then 30 µL of the silicone hardener (component B) was added. The mixture was placed in the desired mold and dried overnight in ambient conditions*.* Devices were fabricated using unmodified 520 nm LED (WINGER^®^ WEPGN1-S1 Power LED Star; 1W) as a pumping source. To carry out the photostability and conversion measurements the color filters were placed either directly on top (referred to as on-chip in BioHLEDs) or at 2 cm distance (referred to as remote in BioHLEDs) of the commercial unmodified LED and measured at different applied currents under ambient conditions. The emission spectra were recorded through an Avantes Spectrometer 2048L (300 VA grating, 200 µm slit, CCD detector) coupled with an AvaSphere 30-Irrad Integrated sphere, monitoring the temperature using a thermographic camera FLIR ETS320. The employed power source was a Keithley 2231-A-30-3. The photometric luminance (cd m^-2^) was measured using a CAS 120 Array Spectroradiometer from Instrument Systems coupled with a TOP150 Telescopic Optical Probe from Instrument Systems (company calibrated).

**Figures**


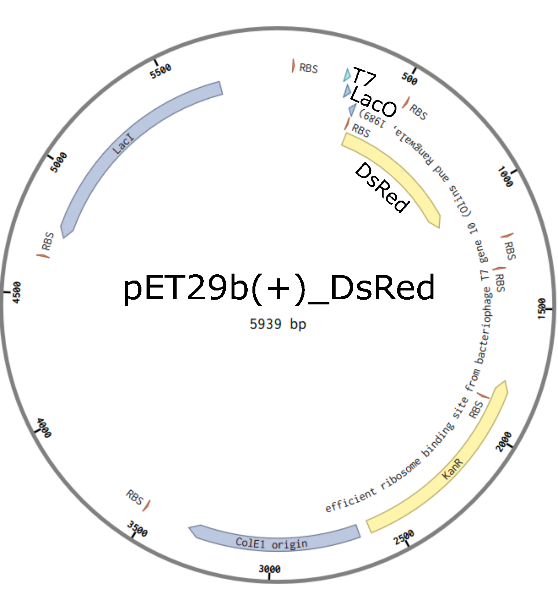


**Figure S1.** The used vector for the expression of DsRed in *E. coli* and *V. natriegens.*

*
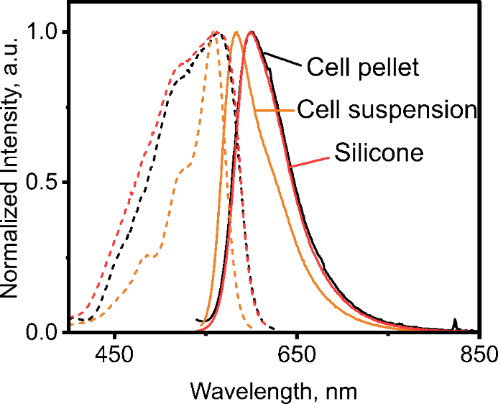
*

**Figure S2.** Emission (solid line; λ_ex_ = 520 nm) and excitation (dashed line; λ_em_ = 650 nm) spectra of pelleted cells, cell suspension in PBS, and hemisphere-shaped silicone filters of *V. natriegens* cells expressing DsRed.


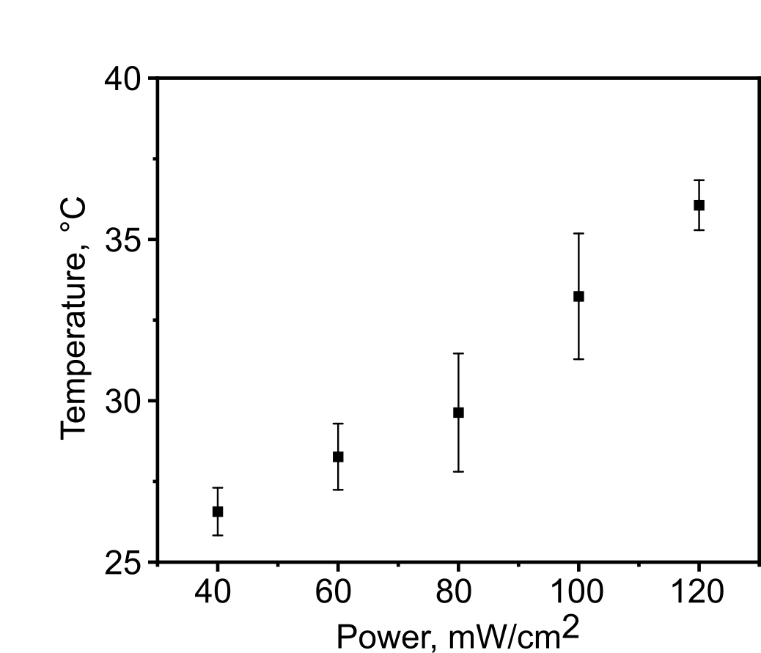


**Figure S3.** Devices were driven at different excitation powers, resulting in an increase of the *V. natriegens*-silicone coatings temperature from 28 °C to 37 °C*.* Three independent replicates were analyzed.


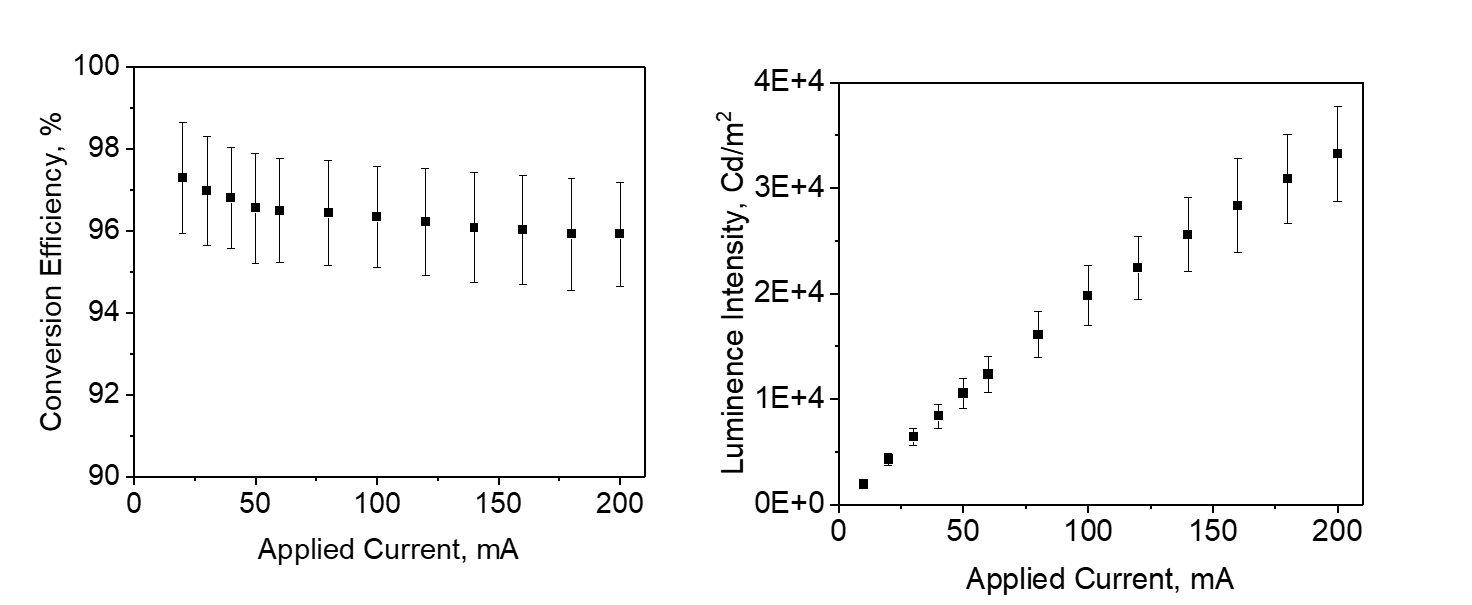


**Figure S4.** Conversion efficiency changes (left) and luminance increase (right) of *V. natriegen*s-silicone devices upon increasing the applied current. Three replicates are shown for statistics.


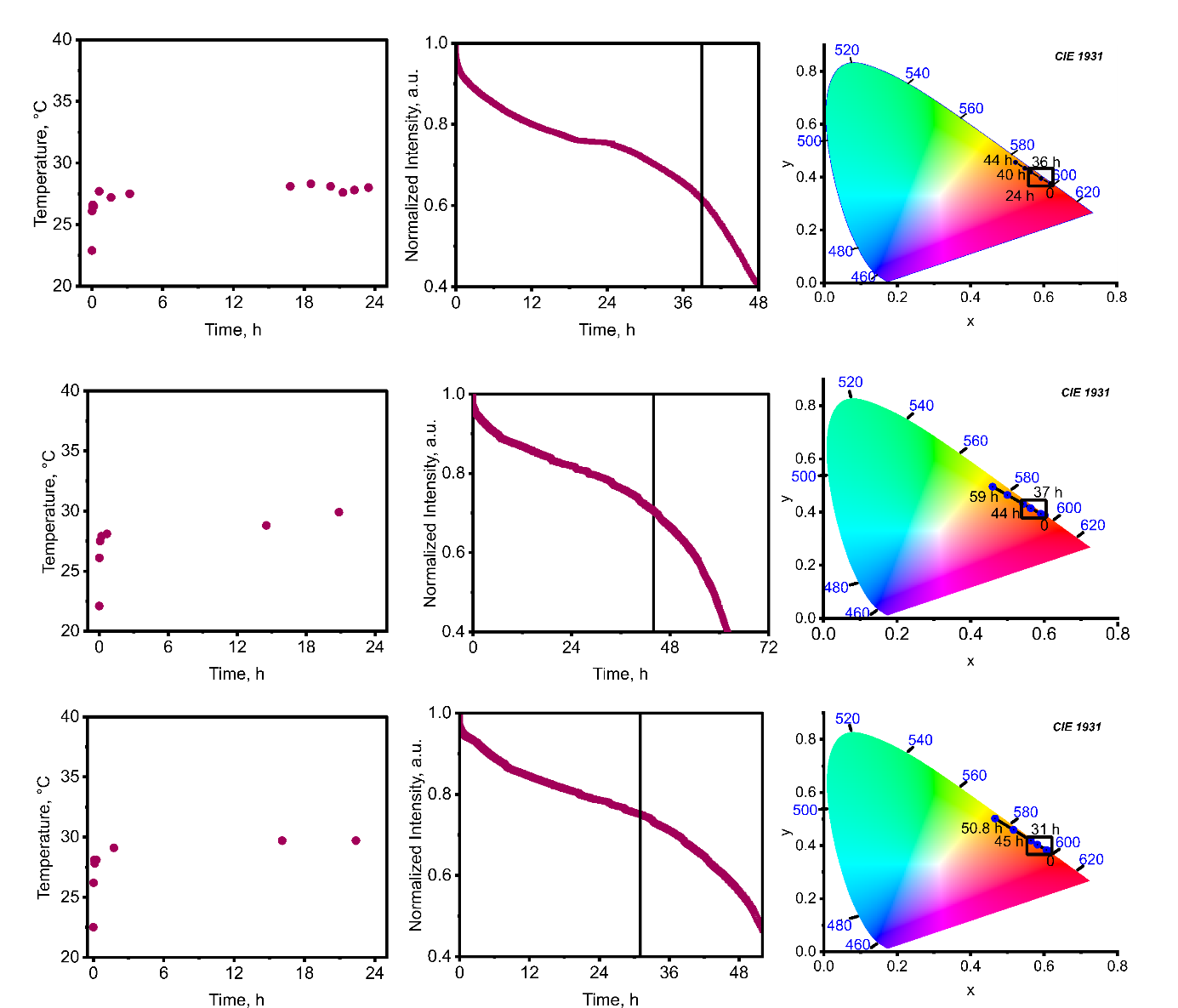


**Figure S5.** Temperature (left), emission intensity decay (center), including the time of the color corruption (black line), and *x/y* CIE color coordinates (right) changes over time of devices operating with representative *V. natriegens* batches as shown for the magenta devices in **Figure 3B**.

**
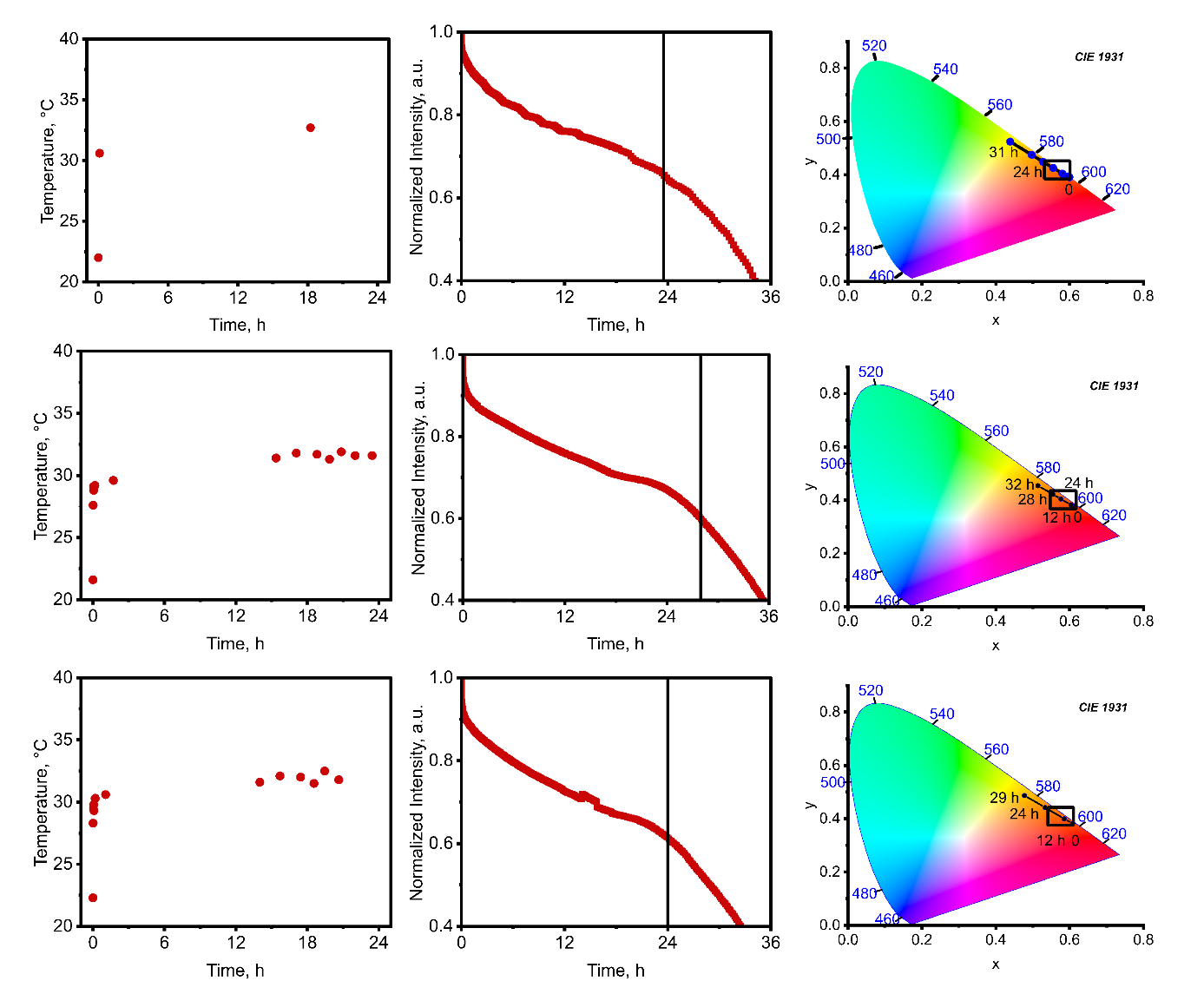
**

**Figure S6.** Temperature (left), emission intensity decay (center), including the time of the color corruption (black line), and *x/y* CIE color coordinates (right) changes over time of devices operating with representative *V. natriegens* batches as shown for the red devices in **Figure 3B**.

**
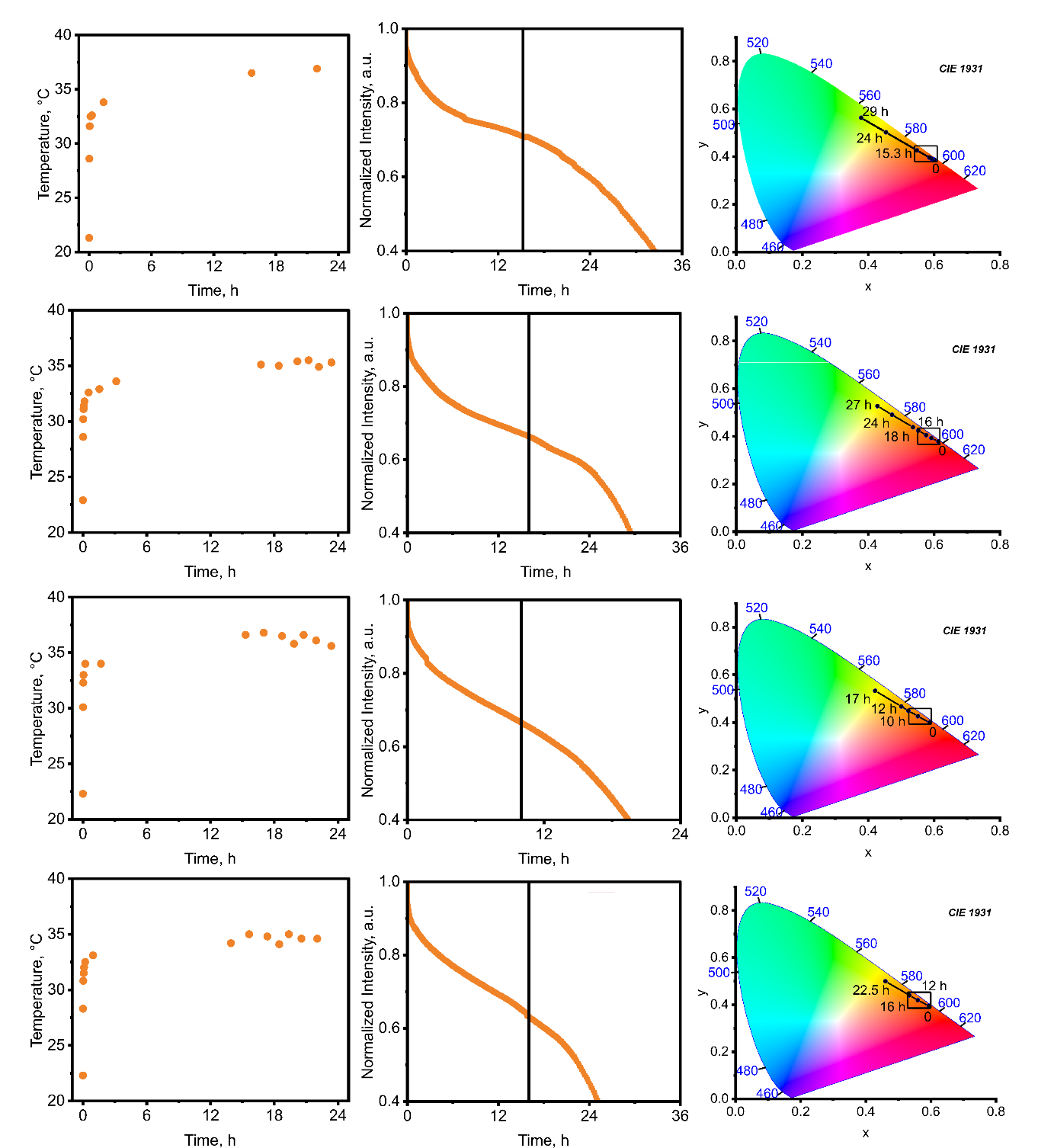
**

**Figure S7.** Temperature (left), emission intensity decay (center), including the time of the color corruption (black line), and *x/y* CIE color coordinates (right) changes over time of devices operating with representative *V. natriegens* batches as shown for the orange devices in **Figure 3B**.


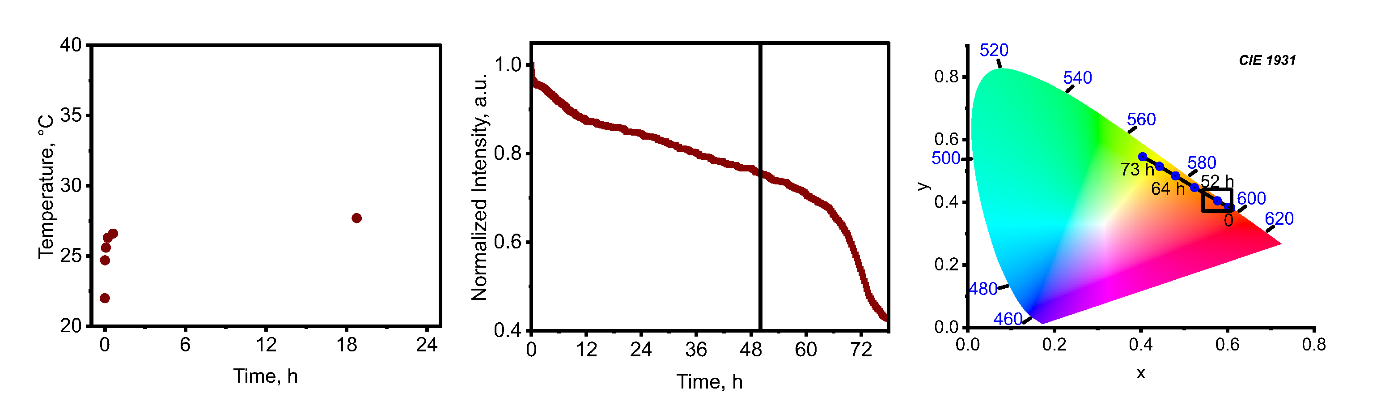


**Figure S8.** Temperature (left), emission intensity decay (center), including the time of the color corruption (black line), and *x/y* CIE color coordinates (right) changes over time of *V. natriegens*-devices operating at the lowest temperature of *ca*. 28 ° C in **Figure 3C**.

**References**

[1] SGI-DNA, Inc, “VMAX^TM^ X2 CHEMICALLY COMPETENT CELLS Instructions,” can be found under https://files.sgidna.com/docs/40029-Vmax-Instructions.pdf, **2020**.

[2] E. Hoffart, S. Grenz, J. Lange, R. Nitschel, F. Müller, A. Schwentner, A. Feith, M. Lenfers-Lücker, R. Takors, B. Blombach, *AEM* **2017**, *83*, e01614.

[3] M. T. Weinstock, E. D. Hesek, C. M. Wilson, D. G. Gibson, *Nat. Methods* **2016**, *13*, 849.

[4] C. Schulze, M. Hädrich, J. Borger, B. Rühmann, M. Döring, V. Sieber, F. Thoma, B. Blombach, *Microb. Biotechnol.* **2023**, *17*, e14277.

[5] A. Sillen, Y. Engelborghs, *Photochem. Photobiol.* **1998**, *67*, 475.
